# Physiological and Transcriptome Responses to Combinations of Elevated CO_2_ and Magnesium in *Arabidopsis thaliana*

**DOI:** 10.1371/journal.pone.0149301

**Published:** 2016-02-16

**Authors:** Yaofang Niu, Golam Jalal Ahammed, Caixian Tang, Longbiao Guo, Jingquan Yu

**Affiliations:** 1 State Key Laboratory of Rice Biology, China National Rice Research Institute, Hangzhou, 310006, China; 2 Department of Horticulture, College of Agriculture and Biotechnology, Zhejiang University, Hangzhou, 310058, Chin; 3 Centre for AgriBioscience, La Trobe University, Melbourne Campus, Victoria, 3086, Australia; Oak Ridge National Laboratory, UNITED STATES

## Abstract

The unprecedented rise in atmospheric CO_2_ concentration and injudicious fertilization or heterogeneous distribution of Mg in the soil warrant further research to understand the synergistic and holistic mechanisms involved in the plant growth regulation. This study investigated the influence of elevated CO_2_ (800 μL L^−1^) on physiological and transcriptomic profiles in *Arabidopsis* cultured in hydroponic media treated with 1 μM (low), 1000 μM (normal) and 10000 μM (high) Mg^2+^. Following 7-d treatment, elevated CO_2_ increased the shoot growth and chlorophyll content under both low and normal Mg supply, whereas root growth was improved exclusively under normal Mg nutrition. Notably, the effect of elevated CO_2_ on mineral homeostasis in both shoots and roots was less than that of Mg supply. Irrespective of CO_2_ treatment, high Mg increased number of young leaf but decreased root growth and absorption of P, K, Ca, Fe and Mn whereas low Mg increased the concentration of P, K, Ca and Fe in leaves. Transcriptomics results showed that elevated CO_2_ decreased the expression of genes related to cell redox homeostasis, cadmium response, and lipid localization, but enhanced signal transduction, protein phosphorylation, NBS-LRR disease resistance proteins and subsequently programmed cell death in low-Mg shoots. By comparison, elevated CO_2_ enhanced the response of lipid localization (mainly LTP transfer protein/protease inhibitor), endomembrane system, heme binding and cell wall modification in high-Mg roots. Some of these transcriptomic results are substantially in accordance with our physiological and/or biochemical analysis. The present findings broaden our current understanding on the interactive effect of elevated CO_2_ and Mg levels in the *Arabidopsis*, which may help to design the novel metabolic engineering strategies to cope with Mg deficiency/excess in crops under elevated CO_2_.

## Introduction

Plants may simultaneously adapted to two or more stresses in their natural ecosystems. Numerous physiological studies have clearly indicated that plant responses to combinations of abiotic stresses represent new modes of stress responses [1–3]. Therefore, it is important to understand the in-depth mechanisms of multiple stress responses by comparing data on single stress with that of combined stresses in order to develop enhanced stress-tolerance strategies in plants. Such studies relevant to agronomically important traits [4] may answer basic questions on signaling “cross-talk” in systems biology [5].

In the past century, the concentration of atmospheric CO_2_ has increased from 318 to 392 ppm; and it is anticipated to exceed 1,000 ppm by the end of the 21^st^ century (cdiac.ornl.gov/pns/current_ghg.html). Nevertheless, increasing concentrations of CO_2_ may have major impacts on plant growth and development [6]. Elevated CO_2_ can be initially beneficial for plant growth [7–11]; however, plant responses to elevated CO_2_ often depend on the availability of soil nutrients [12–14]. Therefore, changes in the nutritional status may greatly influence the results in controlled conditions particularly under elevated CO_2_ which affect plant metabolism and growth.

Excluding H, C and O, magnesium is the fourth most abundant element in plants, after N, K and Ca, Mg is the 8th most abundant mineral element on earth [15]. The level of Mg in the soil is basically maintained by natural genesis and/or fertilization practice and thus both deficiency and excess of Mg should be taken into consideration during developing management strategies. Abnormal Mg status in soil resulting from either Mg depletion or Mg excess is generally considered negative for the growth of the plants [16–23]. Importantly, aside from its crucial role in a vast number of enzymatic reactions including nucleotide metabolism and the turnover of nucleic acids in transcription, splicing or replication, Mg has an additional prominent role as the central atom in the chlorophyll molecules of photosynthesizing organisms [24, 25]. It has been reported that photosynthesis of crop plants depends on its Mg and CO_2_ status in several aspects and both of those resources may have a detrimental effect on plant photosynthesis, depending on the extremity in their levels [7, 26, 27], which eventually result in abnormal or restricted growth of plants [28]. Thus, analysis of elevated CO_2_ and Mg stresses in *Arabidopsis* and other plants revealed that elevated CO_2_ or Mg stress applied separately affects plant biomass, photosynthesis, respiration, root growth and nutrient homeostasis. In spite of their indispensable roles in the central process of photosynthesis and other aspects of the plant, no previous study has investigated the combined effects of elevated CO_2_ and Mg status on plant performance.

In the present study, elevated CO_2_ and Mg availability were selected as the combined target perturbations to unveil possible physiological and molecular alterations in *Arabidopsis* by transcriptome sequencing. Our study brings new insights into interactive effects of elevated CO_2_ and Mg availability at molecular levels.

## Materials and Methods

### Plant materials and growth conditions

The seeds of *Arabidopsis* wild ecotype line (Col-0) were obtained from the Nottingham *Arabidopsis* Stock Centre (http://nasc.nott.ac.uk). All plants were grown in the controlled growth room under a 10 h light/14 h dark photoperiod at constant temperature of 22°C, 80% relative humidity and light intensity of 120 *μ*mol photons m^−2^ s^−1^ as previously described by study of [22].

### Experimental design and setup

The present study was designed with three levels of Mg^2+^ (supplied as MgSO_4_) and two concentrations of CO_2_ in various combinations. Initially, seeds of *Arabidopsis thaliana* genotype Col-0, were surface-sterilized, and germinated on nylon net with proper porosity floating on half-strength Hogland’s nutrient solution. The composition of the culture solution as previously described by study of [10]. Altogether, seedlings were cultured hydroponically for 5 weeks (including germination period) before imposition of CO_2_ and Mg treatments. As CO_2_ treatments, plants were exposed to either ambient CO_2_ (350 ± 50 μL L^−1^) or elevated CO_2_ (800 ± 50 μL L^−1^) conditions. For each CO_2_ condition, plants were divided into three groups: one supplied with 1,000 μM Mg (Control Mg, C), one supplied with 1 μM Mg (Low Mg, L) and one supplied with 10,000 μM Mg (High Mg, H). Concentrations of Mg in the medium were adjusted by manipulating the concentration of MgSO_4_. The solution pH was adjusted to 6.0. The treatment solutions were renewed every 2 d. Ambient CO_2_ + control Mg (AC), ambient CO_2_ + low Mg (AL), ambient CO_2_ + high Mg (AH), elevated CO_2_ + control Mg (EC), elevated CO_2_ + low Mg (EL), elevated CO_2_ + high Mg (EH). Simultaneous treatments of CO_2_ and Mg lasted for 7 days before termination of the experiment.

### Phenotype analysis

Growth parameters were determined in 8 plants per treatment. For the growth analysis, plants were photographed vertically after the 7-d treatments with a high resolution digital camera (Sony RX100, Japan). Then, plants were divided into shoots, roots, each of which was weighed on a precision balance. Both shoots and roots were recorded and quantified for rosette area and leaf area using the public domain image analysis program Image J version 1.43 (http://rsb.info.nih.gov/ij/). The scale was set for the picture within the program. Digital images were captured and processed using Image J. scanned at 300 dpi resolution for measurement of rosette diameter. Leaf number was determined by counting the number of true leaves (>1 mm long leaf blades) per plant.

### Chlorophyll concentration in leaves

Leaf chlorophyll content was determined by using a portable chlorophyll meter (SPAD-502, Minolta, Japan) [9]. Fully expanded leaves were randomly selected from three positions that corresponded to the old, mature, and young parts under different treatments. For each leaf position, 2 SPAD values were randomly collected avoiding main veins during measurement.

### Root growth and morphological analysis

After 7-d treatment, root was scanned to analyze the root morphology (total root length and total number of root tips) using an automatic root scanner (STD1600, Seiko Epson Corp., Japan), and analyzed using the WinRHIZO image analysis software (Regent Instruments, WinRHIZO-EC, Canada). The root-to-shoot ratio was calculated from dry weights of roots and shoots. Additionally, root hairs in 3-cm apical root segments were imaged through a light microscopy with differential interference contrast optics. Micrographs were recorded using a CCD camera (Nikon Eclipse E600).

### Analysis of elemental composition in plant tissue

After 7-d treatment, plants were harvested, washed thoroughly with deionized water, divided into shoots and roots, and dried in an oven at 75°C for 12 h. The samples were then weighed, digested in sulfuric acid/hydrogen peroxide, and analyzed for total P concentration using the vanadium-molybdenum-blue photometric method. For other elements, the dried root and shoot samples were wet-digested in the concentrated HNO_3_/H_2_O_2_ at 90, 120 and 140°C for 2 h, respectively, and then further digested at 180°C until the digest became clear as described by [11]. Concentrations of K, Mg, Ca, Fe, S, Mn and Na in the digests were analyzed by ICP-MS (Inductively coupled plasma mass spectrometer, Agilent 7500a, USA). The concentration of nutrients was calculated on a dry-weight (DW) basis.

### Tissue collection and RNA isolation

For each condition, two samples were collected, each of which was pooled from eight independent plants at the same growth stage. The sampling was done at the start of the light period of treatment (day-7). Total RNA was isolated from shoots and roots using the RNeasy mini kit (QIAGEN, Germantown, MD, USA) with an additional DNase I (QIAGEN) digestion step to remove any genomic DNA contamination. The concentration of the purified RNA was determined by a Qubit2.0 fluorometer (Invitrogen, Carlsbad, CA, USA). RNA integrity was assessed by the Agilent Technologies 2100 Bioanalyzer.

### RNA-Seq

1 μg of total RNA from each sample (n = 2 per treatment) was collected for RNA-Seq library construction and sequencing. cDNA library was constructed using the TruSeq RNA Sample Prep Kit (Illumina, San Diego, CA) according to the manufacturer's instructions after mRNA purification and fragmentation. The samples were then clustered and sequenced on an Illumina HiSeq 2500. Deep sequencing was performed with two replicates for each treatment (twenty-four samples in total) for a 151 cycle pair end run.

### RNA-Seq data analysis

RNA-Seq reads were assessed for quality control with FastQC (version 0.10.1; Babraham Bioinformatics, Cambridge, UK). Reads were mapped to a reference *Arabidopsis* genome (TAIR10, http://www.Arabidopsis.org) using TopHat with parameter (-I 30000) (version 2.08; [29]). The gene abundance values were measured as fragments per kilobase of exon per million fragments per kilobase of exon per million fragments (FPKM) mapped by Cufflinks 2.1.1 [30]. Cuffdiff [31] was then used to determine differential expression (FDR ≤ 0.05). In present study, approximately 6 million pair-end reads from all 24 libraries were trimmed with Sickle and mapped to the *Arabidopsis* TAIR10 genome reference sequences representing 74% transcripts.

### Gene function and pathway analysis

The list of differentially expressed genes generated from Cuffdiff was imported into agriGO tools for Gene Ontoglogy enrichment analysis (http://bioinfo.cau.edu.cn/agriGO/). Biological function and KEGG pathways were determined to be over-represented using the Fisher exact test with a false discovery rate (FDR) correction (FDR≤0.05).

### Extraction of total RNA and quantitative real time PCR (qPCR)

Total RNA was extracted by RNAisoPlus (Takara, Otsu, Shiga, Japan) from about 50 mg of fresh root tissues. Four independent biological replicates were performed on independent root material from different plants. All RNA samples were checked for DNA contamination before cDNA synthesis. cDNA was synthesized, and possible residual genomic DNA contamination was verified as described in our previous study [11]. The mRNA levels of all genes were detected by the Mix SYBR Green RT-PCR kit (Takara, Otsu, Shiga, Japan) with following pairs of gene-specific primers. *UBQ10* was chosen as the housekeeping reference according to [32].

### Statistical analyses

All statistical analyses were performed with DPS software (Stirling Technologies Inc., China). Means were compared by using the *t* test or the Fisher’s least significant difference test at *P* = 0.05 in all cases.

## Results

**Fig 1:**
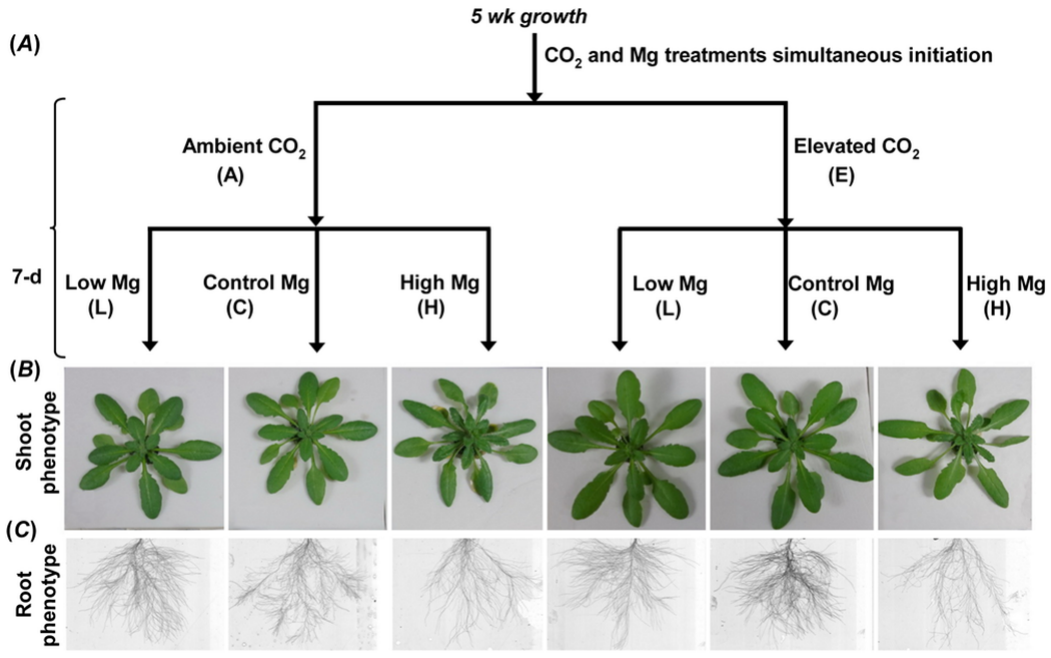
Schematic representation of the experimental design showing all six treatments (individual and combined). Conceptual model (A) and photos of shoots (B) and images of roots (C) of five-week-old wild-type *Arabidopsis* grown for 7 d in low, control and high Mg nutrient solution under ambient or elevated CO_2_. Each arrow corresponds to a treatment; each node corresponds to a physiological state.

A control Mg concentration (1,000 μM) for *Arabidopsis* growth was selected according to the study of [33, 34]. Specially, 10,000 μM MgSO_4_ was selected as a maxmium-level Mg concentration in the present experiment based on our previous study by [22] which showed that *Arabidopsis* plants could sustain in this stress for seven days before severe detrimental effects becoming apparent. Thus, it was certain that within the timeframe of the present study, any observed physiological changes would reflect the response of the plants to the applied treatments without any interference from cellular death effects. Meanwhile, at the harvest, some growth parameters differed among the treatments. For the above reasons, three representative concentration of Mg were chosen to study the interaction with elevated CO_2_ in analysis of transcriptional and metabolic physiology of *Arabidopsis* (Fig 1).

### Morphological and physiological characterization

**Fig 2:**
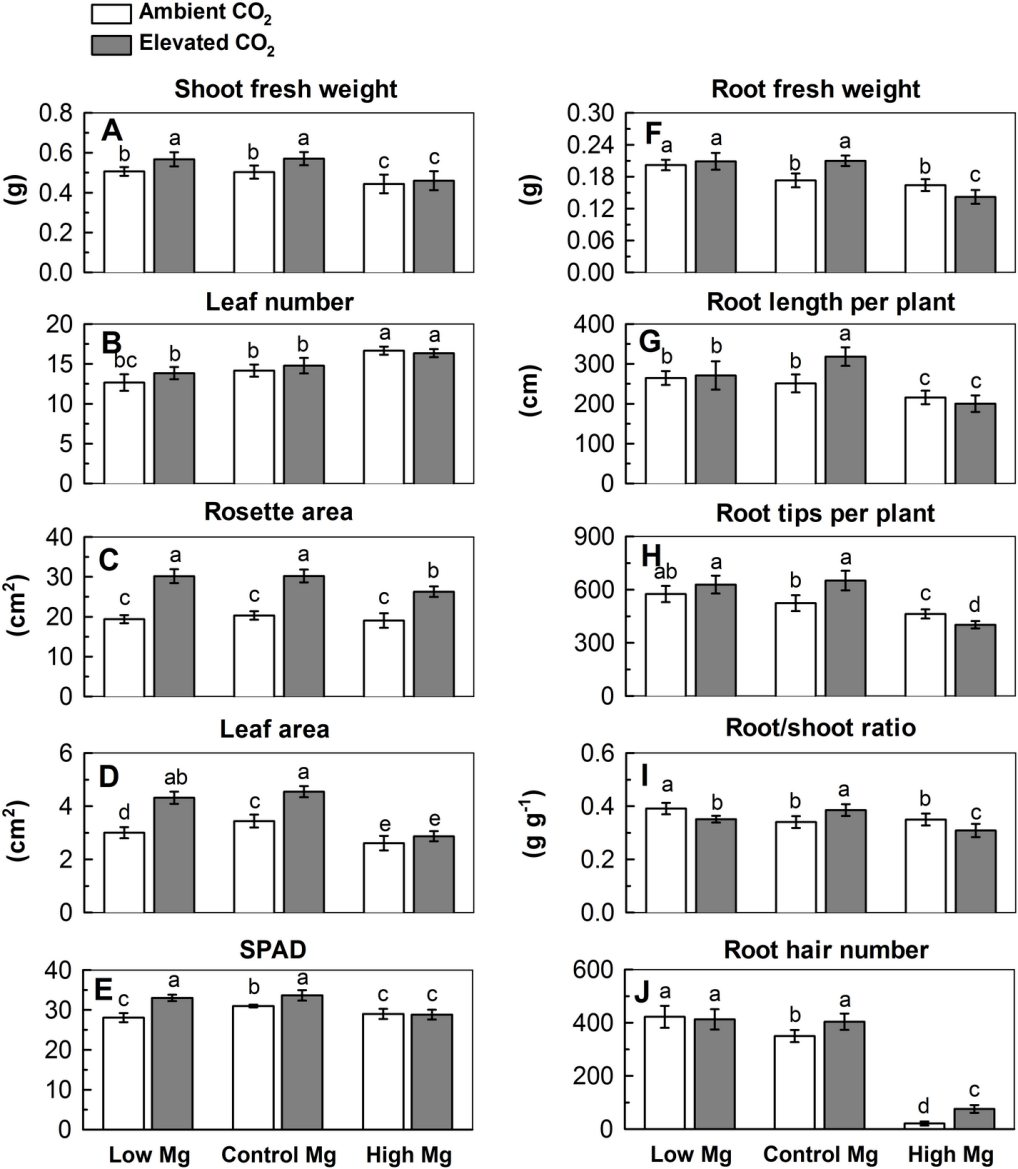
Shoot fresh weight (A), leaf number (B), rosette area (C) and leaf area (D) per plant, SPAD (E), root fresh weight (F), root length (G) and number of root tips per plant (H), root/shoot ratio (I) and number of root hairs per plant (J) of 5-week-old *Arabidopsis thaliana* under six treatments (individual and combined). Means with a same letter within a measurement are not significantly different at *P*≤0.05.

Elevated CO_2_ enhanced shoot growth under low Mg and normal Mg supply, whereas it enhanced root growth under exclusively normal Mg supply (Fig 2). Specifically, the fresh weight, rosette area, leaf area and chlorophyll content (SPAD value) in shoots were higher in EL and EC treatments whereas lower in AH and EH treatments than in the control treatment (Fig 2A and 2C–2E), suggesting that elevated CO_2_ could enhance the growth and chlorophyll content under both low Mg and normal Mg supply. By contrast, compared with the control, number of young leaf was increased in AH and EH treatments but not altered by AL or EL treatment (Fig 2B).

The root fresh weight, total root length, number of root tips per plant, density of root hairs and root/shoot ratio were all greater under EC than under AC condition (Fig 2F–2J), which is in accordance with the results of other studies [9–11]. By comparison, root fresh weight, total root length, number of root tips per plant were increased in the AL and EL treatments but decreased in AH and EH where the decrease was greater in EH than in AH (Fig 2F–2H). It is worth mentioning that the root/shoot ratio was only increased in AL and EC (Fig 2I). Interestingly, the formation of root hairs was completely suppressed by high Mg (10 mM) but was partly restored by elevated CO_2_ (Fig 2J). Overall, these results indicated that elevated CO_2_ enhanced the growth of plant depending on Mg supply and the magnitude of the effect was substantially different in shoots and root tissue.

### The interactive effect of elevated CO_2_ and Mg on mineral homeostasis

**Fig 3:**
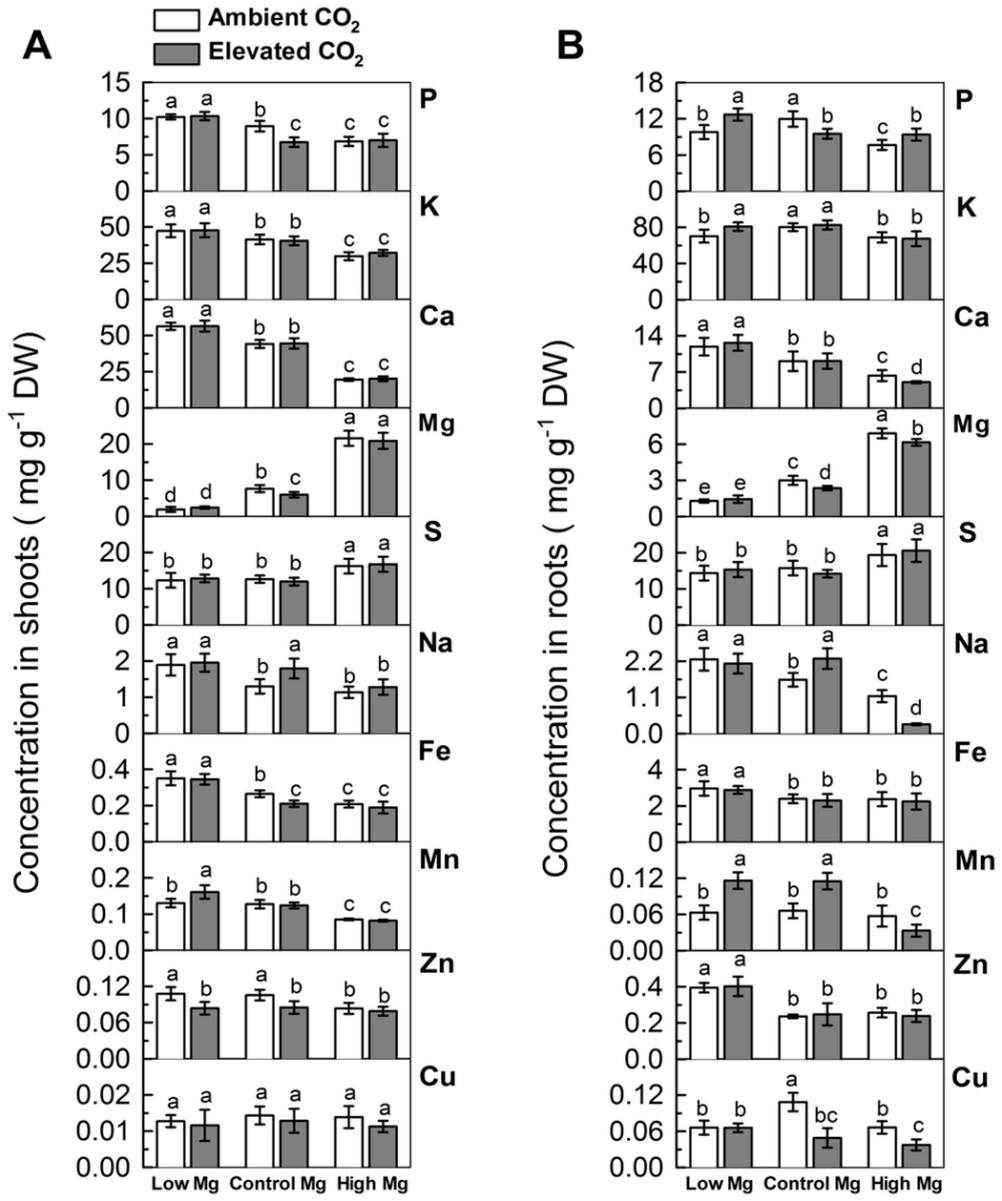
Nutrient composition in shoots (A) and roots (B) of 5-week-old *Arabidopsis thaliana* grown for further 7 d in various CO_2_ and Mg conditions. Values are the averages of at least five samples ± SD. Means followed by a same letter (italics) within a root segment are not significantly different at *P* < 0.05. DW, dry weight.

It is well documented that Mg availability affects the ionome by impacting the uptake and distribution of other cations [19, 20, 35–37]. Meanwhile, analysis of elevated CO_2_ in *Arabidopsis* and other plants revealed that elevated CO_2_ remarkably affects plant biomass and nutrient homeostasis depending on level of nutrient supply [9, 14, 18]. Elevated CO_2_ decreased the concentration of P, Mg and Fe in leaves of normal Mg-supplied plants (Fig 3A). However, with supply of low Mg or high Mg, the plants had a similar level of these elements in shoot under both ambient and elevated CO_2_ treatments. Elevated CO_2_ increased Mn concentration in shoots of the low-Mg-supplied plants but did not alter it in normal Mg or high Mg-supplied shoots. In contrast, elevated CO_2_ decreased the level of Zn in shoots of low Mg and normal Mg-fed plants but did not change it in shoots of plants grown in high Mg treatment. In addition, high Mg decreased the concentrations of P, K, Ca, Fe and Mn while low Mg enhanced the concentrations of P, K, Ca and Fe in a given CO_2_ treatment (Fig 3A).

Similarly, elevated CO_2_ decreased P concentration in roots of the normal Mg-supplied plants but increased it in roots of the low Mg or high Mg-supplied plants. However, elevated CO_2_ decreased the concentration of Ca, Na and Cu roots of high Mg^-^fed plants but did not change that of low Mg-fed plants (Fig 3B). In addition, elevated CO_2_ increased the concentration of K and Mn in roots of low Mg-fed plants. Irrespective of CO_2_ concentration, a general response of roots to low Mg supply is the higher concentration of Ca, Na, Fe and Zn. Regardless of Mg supply, elevated CO_2_ did not affect the absorption of S, Fe and Zn in roots.

### Transcriptome analyses under different CO_2_ and Mg conditions

**Fig 4:**
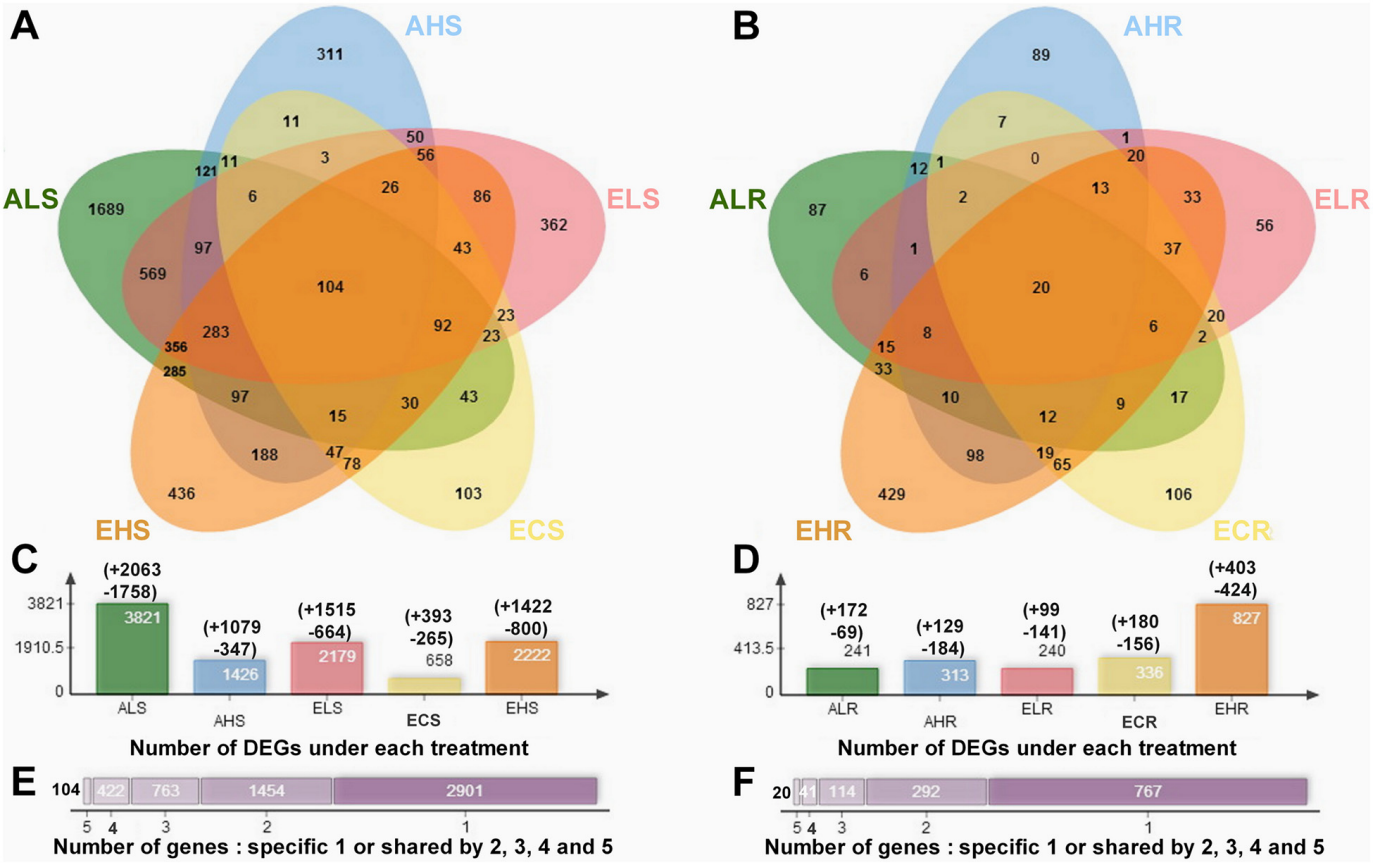
Venn diagrams representing differentially regulated genes ≥ two-fold change (FDR < 0.05) in shoots (A) and root (B) of 5-week-old *Arabidopsis*. Each list in the venn diagram denotes by a transparent shape and overlaps shape indicates elements shared between lists or more often the corresponding counts. Five lists input data of AL, AH, EL, EC and EH were highlighted for both shoots and root in green, blue, pink, yellow and orange accordingly. Number of the total differently regulated genes in shoots (C) and root (D) under each treatment as compared to the control (ACS and ACR, respectively). “+” indicates up-regulation, “-” indicated down-regulation. Some count values are displayed in the chart and some are outside the chart, using lines to line the count to its corresponding area. Common and specific components responded to one or multiple sets in shoots (E) and root (F). 2, 3, 4 and 5-set Venn diagram indicates the output set orders in the resultant extracted datasheet in panel.

The entire data set has been uploaded to the National Institutes of Health Gene Expression Omnibus database (GSE64501) and the raw sequence data have been deposited in the NCBI Gene Expression Omnibus (GEO). A summary of the complete transcriptomic analysis is presented in S1 Table. The Pearson correlation coefficients of sample pairs calculated with gene FPKM, were visualized using heat spectrum graphs where colors ranging from yellow to green correspond to correlation coefficients of 0.5 to 1.0, respectively (S2 Table). To focus on the differently expressed genes (DEGs) under CO_2_ and Mg treatments compared with the control condition, we only selected those with FDR ≤ 0.05. A total of 6345 genes showed ≥ two-fold change in expression upon CO_2_ and Mg treatments (|ΔS| = |log_2_S_X_-log_2_S_Ctrl_| > 1, where X is the CO_2_ and Mg stress treatments) for at least one sample in shoots (Fig 4A) and roots (Fig 4B). 5644 and 1234 genes were identified to be differentially changed in shoots and roots, respectively. And the expression of the down- and up-regulated genes in any treatment groups is also presented in S3 Table.

Venn diagrams showed that there are 3,821 (AL/AC), 1,426 (AH/AC), 2,179 (EL/AC), 658 (EC/AC) and 2,222 (EH/AC) DEGs in shoots (Fig 4A). Meanwhile, as compared with the control, 1689, 311, 362, 103 and 436 DEGs was specifically respond to ‘AL’, ‘AH’, ‘EL’, ‘EC’ and ‘EH’ treatment, respectively (Fig 4A). There are 569 and 188 common differential expressed genes in shoots between ‘AL vs EL’ and ‘AH vs EH’, respectively (Fig 4A). Furthermore, the maximum and minimum DEGs were recorded in shoots of plant grown in AL and EC treatments, respectively (Fig 4C). It is suggested that low Mg had a greater while elevated CO_2_ had a smaller effect on transcript responses in shoots.

In roots, 241 (AL/AC), 313 (AH/AC), 240 (EL/AC), 336 (EC/AC) and 827 (EH/AC) genes altered their expression under combined treatments of CO_2_ and Mg (Fig 4B). As compared with the control, there were 87, 89, 56, 106 and 429 differentially expressed genes in roots under ‘AL’, ‘AH’, ‘EL’, ‘EC’ and ‘EH’, respectively (Fig 4B). In contrast with shoot, elevated CO_2_ had a few affect transcripts in the roots with low Mg (20% of variance) but transcripts in the roots with high Mg were more prominent under elevated CO_2_ (67% of variance) than under ambient CO_2_ (25% of variance). Moreover, there are only 6 but 98 common differential expressed genes in roots between ‘AL vs EL’ and ‘AH vs EH’, respectively (Fig 4B). The maximum and minimum DEGs were recorded in shoots of plant grown in the EH and EL treatments, respectively (Fig 4C and 4D).

**Fig 5:**
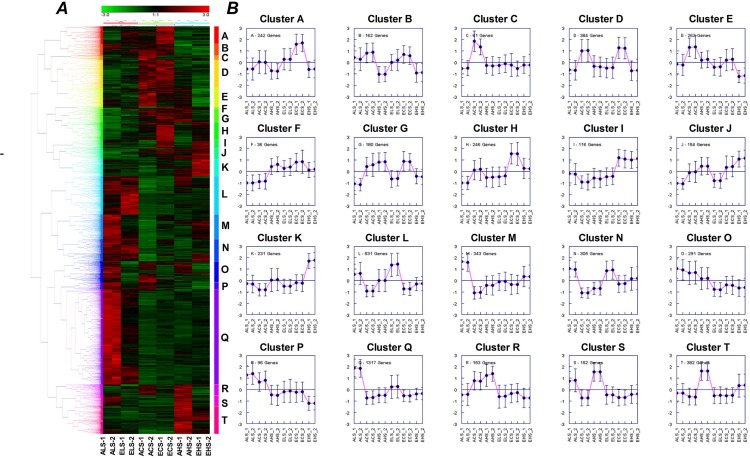
Hierarchical document clusters (A) and their corresponding expression patterns (B) in 12 different shoot samples under CO_2_ and Mg treatments using average linkage llustering and euclidian distance. For each treatment, two admixture biological samples were performed on independent root material from ten different plants. Each column represents a single sample. Data adjustment procedures are employed and often used prior to statistical analysis of a given data set. The color scale ranges from saturated green for log ratios -3.0 to below saturated red for log ratios 3.0 and above. Each gene is represented by a single row of colored boxes; Cells with log ratio of 0 (genes unchanged) are colored black, increasingly positive log ratios with reds of increasing intensity, and increasing negative log ratios with greens of increasing intensity. Missing values usually appear gray. Gene expression is plotted on a log_2_ scale for each gene transcript and details about individual transcripts can be found in Supporting Information S3 Table.

**Fig 6:**
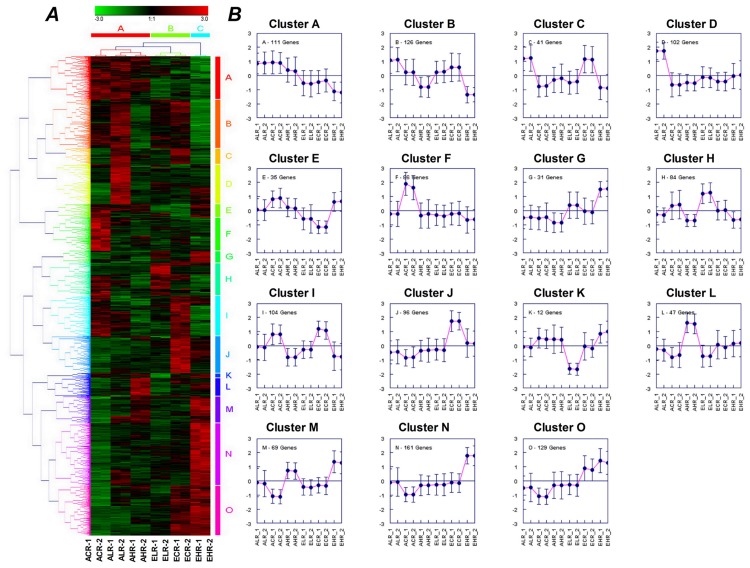
Hierarchical document clusters (A) and their corresponding expression patterns (B) in 12 different root samples under CO_2_ and Mg treatments using average linkage llustering and euclidian distance. The color scale ranges from saturated green for log ratios -3.0 to below saturated red for log ratios 3.0 and above. Each gene is represented by a single row of colored boxes; Cells with log ratio of 0 (genes unchanged) are colored black, increasingly positive log ratios with reds of increasing intensity, and increasing negative log ratios with greens of increasing intensity. Missing values usually appear gray. Gene expression is plotted on a log_2_ scale for each gene transcript and details about individual transcripts can be found in Supporting Information S3 Table.

There are nearly half of differently expressed genes in shoots were shared by more than two treatments (Fig 4E and 4F), revealing high overlap of transcript responses between elevated CO_2_ and Mg stresses treatments. We further classified all expression plots of DEGs into 20 (A-T) and 15 (A-O) clusters in shoots (Fig 5) and roots (Fig 6), respectively, according to genes expression patterns using hierarchical clustering with complete linkage. Several data adjustment procedures are available and often used prior to statistical analysis of a given data set [38]. Gene expression is plotted on a log_2_ scale for each gene and details about individual genes can be found in Supporting Information S3 Table. It is noticed that clustering results of treatments showed a completely different pattern between shoots and roots. In shoots, the DEGs was clustered into three groups according to the concentrations of Mg (Fig 5) while in roots it was clustered into two groups according to CO_2_ treatments (Fig 6).

### Validation of gene expression profiles using qPCR

To evaluate the accuracy of expression profiles obtained from RNA-Seq, we first measured a selected set of DEGs genes by real-time qPCR, using the same samples originally used for RNA-Seq (S4 Table). We analyzed the transcript levels of nuclear-encoded genes related to photosynthesis, phosphorus-containing anhydrides, cell wall, stress response, root development and auxin pathway by real-time qPCR. This analysis revealed a close correlation between the expression changes (fold difference) measured by each method (Pearson’s correlation coefficient *r* = 0.90).

### Remarkable responses of genes in shoots

**Fig 7:**
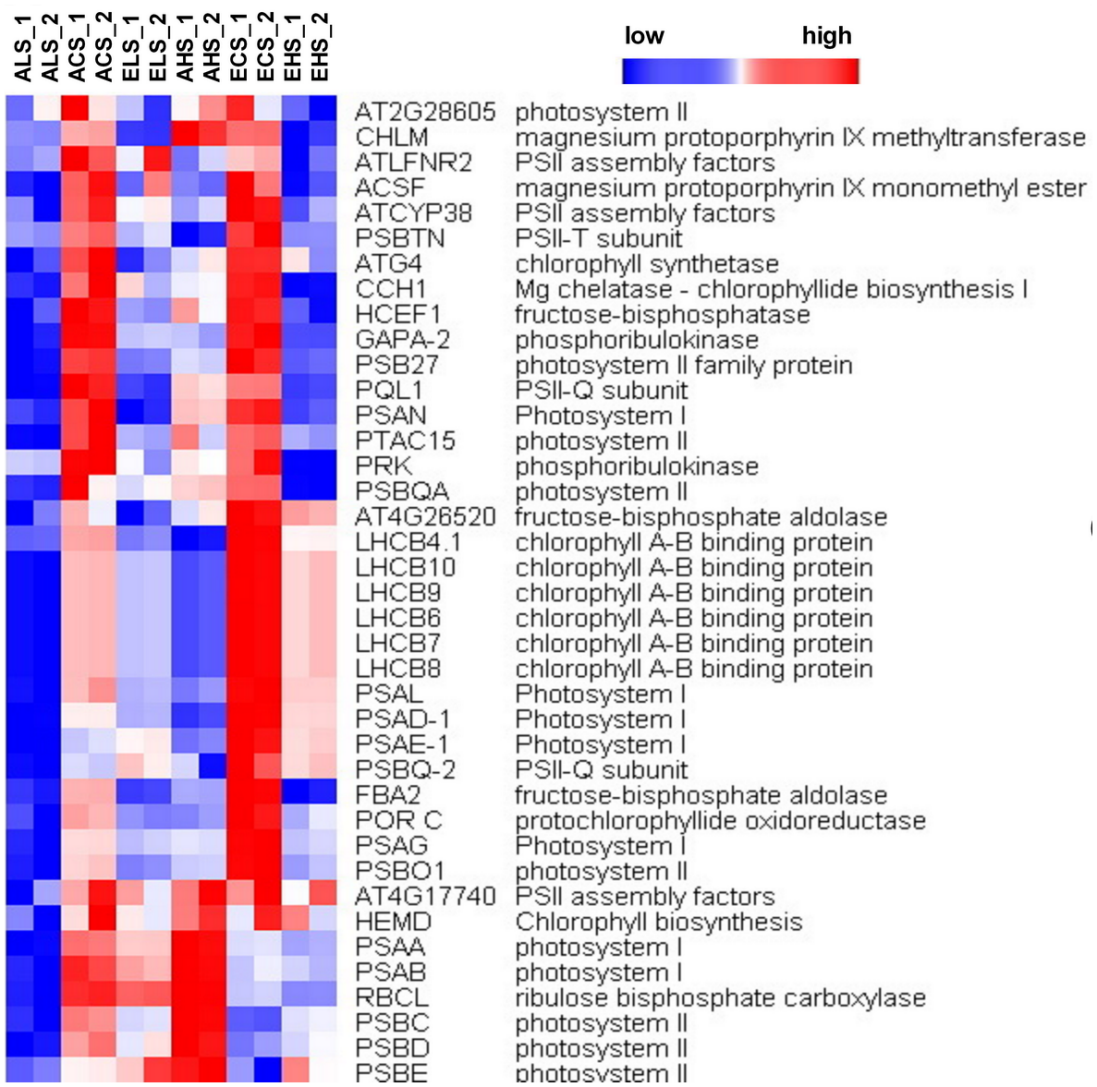
Clustering of transcripts in the category ‘‘photosystem functions” and ‘‘chlorophy II control”. Regulation of transcripts in this category, with sub-categories identified, for six treatments as follows: ALS, ACS, AHS, ELS, ACS and AHS. The figure shows distinct clusters of expression patterns within the group of a treatment across the six treatments. The full cluster set is shown on the left; specific gene names are expanded to the right to allow closer inspection of the differential expression patterns. Higher levels are represented with color red of increasing intensity, and lower levels are represented with blue of increasing intensity. The increased or reduced expression is relative to the mean expression. All transcript levels shown were statistically significant (*FDR* ≤ 0.05) increase or decrease at any one treatment.

Functional enrichment analysis using agriGO for all DEGs is able to reveal biological functions based upon DEGs [39]. The full functional annotation analysis is provided in supplementary S5 Table. We then investigated whether transcripts of the particular response modes could be associated with biological functions via their corresponding, significant GO terms. Firstly, it is found that the top ranked biological functions in the shoots included the most important and significant differences compared with the whole genome representation were in the response to ‘stimulus or stress’, the ‘cell wall type and organization’, the ‘antioxidant and oxidoreductase activity’, the ‘cellular metabolic and multi-organism process’, ‘plastid chloroplast, chloroplast part, chloroplast thylakoid’, ‘membrane part’ and ‘response to hormone pathway’ (S5 Table). Moreover, it is revealed that the strong common existence of genes involved in various treatments of CO_2_ and Mg with approximately 35% and 25% of the total functional annotations categorized as being cell part and stress/ stimulus-associated, respectively. A significant number of these DEGs were affected in more than four of the treatments, and the overlapping sets of genes are over-represented for C, K, and T clusters (Fig 5). Among the differently regulated gene functions with cell part, chloroplast and thylakoid, endomembrane system and cell wall were the maximum ranked cellular functions (Fig 7).

Under normal Mg supply, the significant differences in gene abundance in shoots between ambient and elevated CO_2_ had mainly focused on A and H clusters, respectively (Fig 5). In addition, genes in L cluster specifically respond to interaction of elevated CO_2_ and low Mg. This cluster disease resistance protein mainly included a disease-resistance proteins belonging to the TIR-NBS-LRR (Toll/Interleukin1 receptor–nucleotide binding site–leucine-rich repeat) domain signatures and genes encoding proteases and virulence-responsive proteins. On the other hand, genes in Q cluster related to cadmium-ion response (GO: 0046686), cell redox homeostasis (GO: 0045454) and lipid localization was specifically up-regulated by low Mg (Fig 5 and S6 Table). Most of the features of known genes (such as *ATTRX5*, *RPT2A*, *GDH2*, *PAG1*, *ADL1E*, *NDPK1*) in response to cadmium ion are thought to be cytosolic or associated with the plasma membrane. By comparison, elevated CO_2_ + high Mg had a weak impact on the response of transcripts in shoots. The only interaction between them was enriched in K cluster, which included chloroplast envelope (*AOC1*, *AOC2*, *COR15B*, *LOX2*, *AT3G22620* etc) and monooxygenase activity genes. GO analysis showed that all those six monooxygenase activity genes were also associated with endomembrane system (Fig 5 and S6 Table). Other cluster genes GO term, such as response to hormones and water deficit, were shared between transcripts under EL and EH treatments.

### Functional enrichment in roots

The functional categories most significantly enriched in the roots compared with the whole genome representation were in the response to ‘stimulus or stress’, the ‘cell wall type and organization’, the ‘antioxidant and oxidoreductase activity’, the ‘activity of structural molecule, electron carrier, transporter, catalytic’, the ‘endomembrane system’ and the ‘external encapsulating structure structural’ (S5 Table). This is in accordance with the findings of other studies [18–20]. Among these, a common set of genes responding to ‘stress or stimulus’, ‘cell wall type’ and ‘oxidoreductase activity’ were identified in both of low and high Mg supply irrespective of CO_2_ concentration, and were also shared with the response to shoot. Numerous up-regulated genes in cluster M, N, and O functional enrichment including: cytokinin-mediated signaling pathway, transferase activity, cell wall macromolecule biosynthetic process, water channel activity, rhythmic process, flavonoid metabolic process were found to be specifically located in the combined treatment of elevated CO_2_ and high Mg supply (Fig 6 and S6 Table).

**Fig 8:**
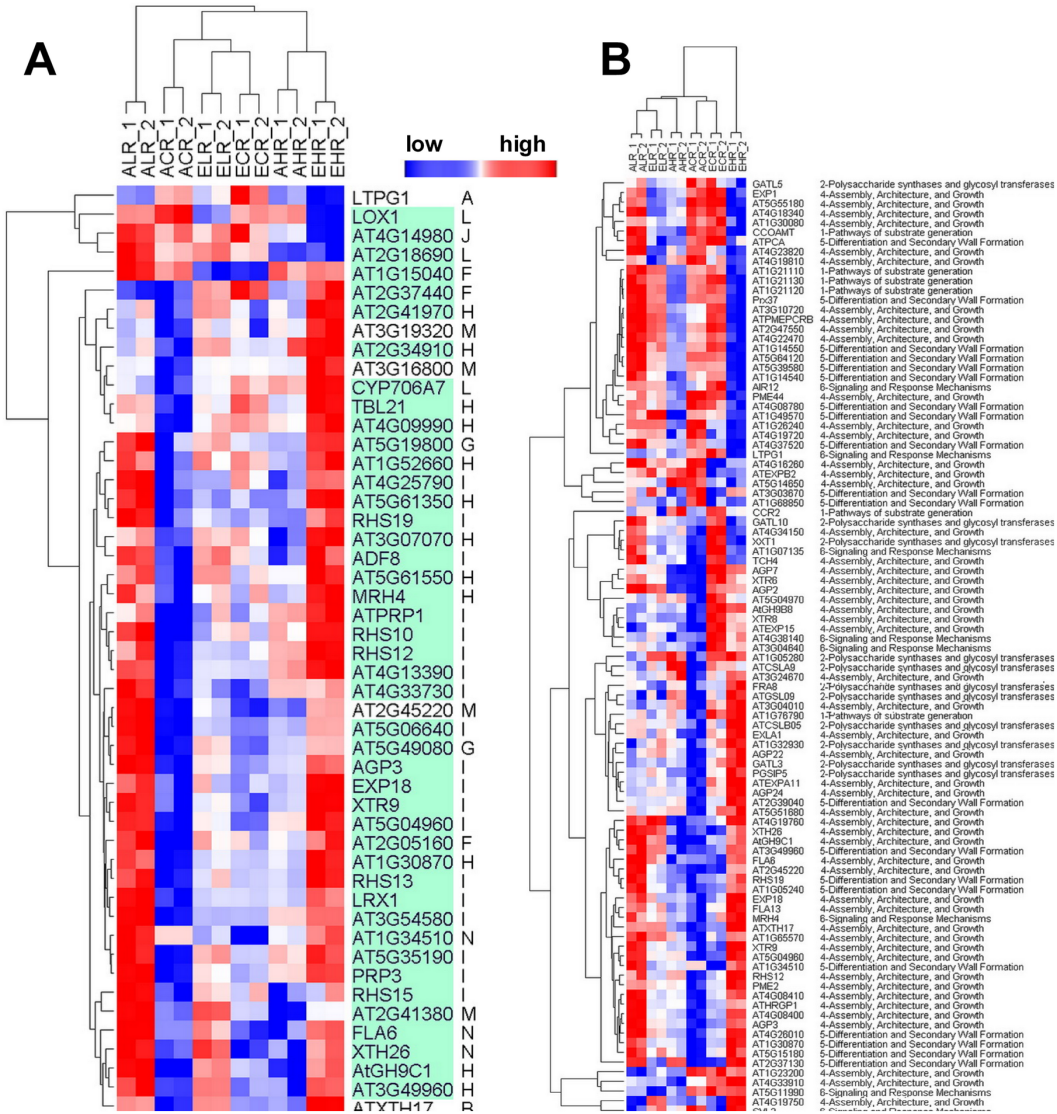
Hierarchical clustering differently regulated genes from the 208 core root epidermal genes (A) and from cell wall gene family (B) based on their relative transcript accumulation in different CO_2_ and Mg treatments. The order of RNA-seq samples along the x-axis is as follows: ALR, ACR, AHR, ELR, ACR and AHR. The figure shows distinct clusters of expression patterns within the group of a treatment across the six treatments. The full cluster set is shown on the left; specific gene names are expanded to the right to allow closer inspection of the differential expression patterns. Red color indicates high transcript level; Blue color indicates low transcript level. The 208 genes are highlighted in green (for root-hair genes).

The EH treatment induced specific genes in roots mainly focused in cluster N (Fig 6). These cluster genes functioned with lipid localization, cell wall and endomembrane system, and specifically increased heme binding (S6 Table). Notably, GO terms showed that all six genes *AT5G46890*, *AT5G46900*, *AT3G22570*, *AT4G12510*, *AG4G12520* and *AT4G22490* related to lipid localization were found encoding protease inhibitor, a lipid transfer protein (LTP) family (Fig 6A and 6B). In contrast, low Mg supply alone elevated a number of up-regulated genes in cluster D related to ‘trichoblast differentiation, epidermis development, root morphogenesis, structural constituent of cell wall, ion homeostasis and transport’. In addition, under both low Mg and high Mg conditions, some DEGs were elevated in those involved in immune system functions, in particular, functions related to cell death, and toxin catabolic and metabolic process and defense response (Fig 8 and S6 Table).

## Discussion

The objective of this work is to understand responses in plants along with interactive effect of elevated CO_2_ and Mg levels by comparing data on single stress with that of combined stresses. Some remarkable transcriptomic responses to individual vs. combinatorial treatments of elevated CO_2_ and Mg levels were highlighted here. Contrasting changes were found between roots and shoots with the shoot transcriptome being more severely affected by low Mg while the root transcriptome more affected by high Mg. Elevated CO_2_ had a greater effect on transcript response in low Mg-fed shoots as well as in high Mg-fed roots.

### Elevated CO_2_ affects plant responses to Mg supply

The increasing interest has been on studying the effects of CO_2_ level on plant responses either phenotypically [40, 41] or transcriptomically [42, 43] and genome-scale studies of the model species *Arabidopsis* under Mg deficiency [19–20] and excess [21, 22]. Here, by analysis of individual vs. combinatorial effect of elevated CO_2_ and Mg levels on phenotypical or transcriptomic responses in *Arabidopsis thaliana* grown in hydroponic cultures (Fig 1), this study showed that elevated CO_2_ affected *Arabidopsis* growth and the response of transcripts to Mg supply and that the magnitude of the effect differed between shoots and roots. The resulted indicated that elevated CO_2_ significantly mitigated the negative impact of low Mg, as apparent in biomass reduction, photosynthesis inhibition, chlorophyll fluorescence decline (Fig 2). This seems suggested that elevated CO_2_-mitigation of low Mg stress associated with maintenance of positive carbon balance and carbohydrate accumulation in *Arabidopsis*. Besides, elevated CO_2_ promoted the root/shoot ratio in normal-Mg-fed plants but inhibited it in both low Mg and high Mg-fed plants (Fig 2I). CO_2_ exacerbated the negative effect of high Mg on root biomass and number of lateral roots but alleviated the effect on root hair density (Fig 2F and 2G). It has been reported that Mg deficiency and elevated CO_2_ changed sugar partitioning between shoot and roots, leading to an increase in the root: shoot biomass ratio [36, 44, 45]. Elevated CO_2_ enhanced both shoot and root growth of normal Mg-supplied plants (Fig 2F–2J). This is in accordance with the results of many studies [9, 11, 46]. Overall, these results indicate that elevated CO_2_ enhanced the growth of plant depending on Mg supply and the magnitude of the effect was similar in shoots and roots. Interestingly, leaf number was increased only in AH and EH treatments but not altered by AL or EL treatment (Fig 2B), indicating that high Mg could induce differentiation and division of leaves regardless of elevated CO_2_. Interestingly, phenotypes of narrow and thick leaves were observed in high Mg-fed leaves under ambient and elevated CO_2_. It is reported that *WUSCHEL-RELATED HOMEOBOX1* (*WOX1*) acts for cell proliferation in the blade outgrowth and margin development downstream of adaxial/abaxial polarity establishment in *Arabidopsis* leaves [47]. In present study, expression of *WOX1* in leaves was greatly decreased by AH and EH treatment (S1 Table) indicating that the *WOX1* are required for promoting cell proliferation in outgrowth of leaf blade under high Mg, maybe due to activated cell division.

However, the responses of transcripts coincided with measurable root parameters such as root weight and root hair number in AL and EL treatments, while the transcripts in roots treated by AH and EH were found not completely concomitant with their final morphological root phenotype (Fig 4 and S6 Table). These suggest that the molecular and physiological responses to low Mg and high Mg conditions differ not only in the number of genes or extents of expression changes, but also in the sets of genes induced.

### Ionomic adjustment in response to elevated CO_2_ and Mg level

The current study showed that the EL increased the concentration of Mn and decreased the concentration of Zn in shoots (Fig 3A) while the EH decreased and the AH did not affect the concentration of Mn in roots (Fig 3B). These results were probably due to the competition of Mn^2+^ with Mg^2+^ for membrane transport and substitute Mg^2+^ for activating a number of enzymes (like ribulose-1,5-bisphosphate carboxylase/oxygenase) [20] because Mn^2+^ and Mg^2+^ have similar chemical properties. This study also indicates that high Mg supply had a profound impact on the ionic balance, noticeably by decreasing the concentration of macro-nutrients including P, K and Ca in both shoots and roots irrespective of CO_2_ treatment. Notably, the enhanced shoot and root growth and decreased concentrations of P and Mg in shoots and roots, Fe in shoots and Cu in roots of normal-Mg-supplied plants by elevated CO_2_ are in accordance with the findings of other studies [9, 11, 14]. The observed decrease in nutrient concentrations is most likely due to a dilution effect caused by enhanced biomass production at elevated CO_2_ [48].

Many transporters involved in metal ion homeostasis have been identified in the *Arabidopsis* genome [49–50]. It is worth mentioning that Mg deficiency did not induce the expression of genes encoding permeases potentially mediating Mg transport, such as the *MITOCHONDRIAL RNA SPLICING2/MAGNESIUM TRANSPORTER* (*MRS2*/*MGT*/*CorA*) family [51–53] and *MAGNESIUM/PROTON EXCHANGER 1*(*MHX*) [54–55]. One of the categories containing the highest number of Mg-regulated ion channel genes was the ‘anion channel protein family, KCO5 protein family, shaker family, glutamate receptor family’. Analysis revealed the existence of genes involved in various treatments of CO_2_ and Mg with approximately 80% of the ion binding functional annotations categorized as being P, S, Ca and Zn-associated.

### Elevated CO_2_ mitigated against low Mg-induced photosynthesis-inhibition

As reported in this and previous studies [26, 28], both Mg deficiency and oversupply have detrimental effects on plant photosynthesis, and consequently resulting in restricted growth of plants. Transcripts of genes involving photosynthetic systems I (e.g. *PSAN*, *PSAL*, *PSAD-1*, PSAE-1, *PSAG*, *PSAA* and *PSAB*) and II (*PSBO 1*, *PSBC*, *PSBD* and *PSBE*) and photosystem II-Q (PSII-Q) and PSII-T subunits were significantly reduced by AL treatment (Fig 7). Moreover, the increased chlorophyll concentration by EL and EC but decreased by AL, AH and EH (Fig 2A and 2C–2E) indicate that low Mg and high Mg restrains the photosynthesis correlated with chlorophyll content. The up-regulation of photosynthesis-related genes in EL relative to AL indicates that elevated CO_2_ alleviated the effects of low Mg. This suggested that plant responses to combinations of elevated CO_2_ and low Mg stress induces a new response of photosynthetic. Mg plays a fundamental role in phloem export of photosynthates, so that Mg deficiency restricts the partitioning of dry matter between roots and shoots, which result in an accumulation of sugars, starch and amino acids in leaves, chlorophyll break-down, an over-reduction in the photosynthetic electron transport chain and the generation of excessive reactive oxygen species (ROS) impairing photosynthetic CO_2_ fixation [56–59]. Evidence for the involvement of the light-harvesting chlorophyll a/b-protein complex in thylakoid stacking and for effects of Mg^2+^ was reported by [60]. Low Mg decreased but elevated CO_2_ restored the expression of genes correlated with chlorophyll a/b-protein and starch biosynthesis. These genes include *SHOOT APICAL MERISTEM ARREST 1* (*SHA1*), *ATP SYNTHASE DELTA-SUBUNIT GENE* (*ATPD*), *THIAMINC* (*THIC*), *SIGMA FACTOR 4* (*SIG4*), *PHOTOTROPIN 2* (*PHOT2*), *NDR1/HIN1-LIKE 12* (*NHL12*), *HIGH CYCLIC ELECTRON FLOW 1* (*HCEF1*), *PROTON GRADIENT REGULATION 3* (*PGR3*), *GLYCINE DECARBOXYLASE P-PROTEIN 1* (*AtGLDP1*) and *THIOREDOXIN F-TYPE 1* (*TRXF1*). Specifically, EL significantly increased the concentrations of homoserine, methionine and β-alanine.

### Defense response upon a combined stresses of elevated CO_2_ and Mg

Interaction of elevated CO_2_ and low Mg specifically triggers disease-resistance proteins mainly including those belonging to the TIR-NBS-LRR (Toll/Interleukin1 receptor–nucleotide binding site–leucine-rich repeat) domain signatures and genes encoding proteases and virulence-responsive proteins. In the plant immune system, it has been shown that NBS-LRR disease-resistance proteins can monitor the homeostasis of type III effector targets [61–63]. On the other hand, elevated CO_2_ intensified the repression of the expression of genes induced by low Mg in Q cluster, which is related to the Cd response (GO:0046686), cell redox homeostasis (GO: 0045454) and lipid localization. Most features of known genes (such as *ATTRX5*, *RPT2A*, *GDH2*, *PAG1*, *ADL1E*, *NDPK1*) responding to cadmium ion are thought to be cytosolic or associated with the plasma membrane.

Compared with the ACS, a larger number of genes functionally responding to programmed cell death and lipid metabolism (e.g. *FADB ATFAH2*, *WAX2*, *FATTY ACID DESATURASE 2*, *FATTY ACID DESATURASE 7*, *CYTIDINEDIPHOSPHATE DIACYLGLYCEROL SYNTHASE 5* and *QUIRKY*) in shoots were suppressed by AL, but were not affected by EL treatment (S3 and S5 Tables).

Interestingly, elevated CO_2_ plus high Mg had a weak impact on the response of transcripts in shoots. Their only interaction is intensified in K cluster, which includes chloroplast envelope (*AOC1*, *AOC2*, *COR15B*, *LOX2*, *AT3G22620* etc) and monooxygenase activity genes. GO analysis showed that all those six monooxygenase activity genes are also associated with the endomembrane system. Not surprisingly, other genes GO term, such as response to hormones and water deficit, are shared between transcripts under AH and EH treatments.

### Mg deficiency affects the root transcriptome irrespective of CO_2_ treatment

The present study and previous transcriptomic analysis of [19–20] suggest that low Mg affects the root development less than the shoot development in *Arabidopsis*. This was clearly distinct from reports on N, P and K, which have a severe impact on the root transcriptome and eventually on root development [44, 64–67]. Phenotype data showed that AL and EL increased root growth and proliferation (Fig 2). At the transcriptional level, low Mg increased but high Mg decreased cell wall synthesis and root hair cell differentiation (e.g. *ATXTH17*, *ATXTH 26*, *LEUCINE-RICH REPEAT/EXTENSIN 1*, *EXORDIUM*, *PRP3*, *POLYGALACTURONASE INHIBITING PROTEIN 1* and *GLYCINE-RICH PROTEIN 5*) (Fig 6). Compared with the controls, there are 87 genes were specifically changed in low Mg^2+^-treated roots, and most of these genes involved in plant-type cell wall organization and oxidoreductase activity were greatly up-regulated in roots of the plant grown in AL treatment (S6 Table). This indicates that low Mg promoted root-hair growth, probably through the characterized ROS signals and the expression of plant-type cell wall genes. The result is consistent with our previous finding [22]. Similarly, under supply of low Mg plus elevated CO_2_, 56 genes were specifically changed in low Mg^2+^ plus CO_2_ treated roots. The expression of low-Mg-induced DEGs in response to stress and stimuli, as well as the activity of cell wall and oxidoreductase, was still up-regulated by high CO_2_ concentration, but there was a large portion of gene expression reinstated as that in control, suggesting that increased CO_2_ level can alleviate the low Mg-induced stimuli-responsiveness and high expression of cell wall genes (Fig 8). Moreover, the up-regulation of cation-binding genes at a low Mg^2+^ plus elevated CO_2_ implies that increased CO_2_ concentration would facilitate the absorption of cations by low Mg-fed roots. On the contrary, elevated CO_2_ did not affect the expression of genes correlated with localization and transport in low Mg-supplied roots and suppressed the activity of ion transmembrane-transporters, the cell metabolism (metabolic processes of fatty acids), and metabolisms of benzene and its derivatives as well as a series of metabolic processes of organic acids, suggesting that the combination of low Mg^2+^ and elevated CO_2_ induced a unique response in the root system of *Arabidopsis thaliana*, particularly having an inhibitory effect on metabolic processes in root cells.

## Conclusion

**Fig 9:**
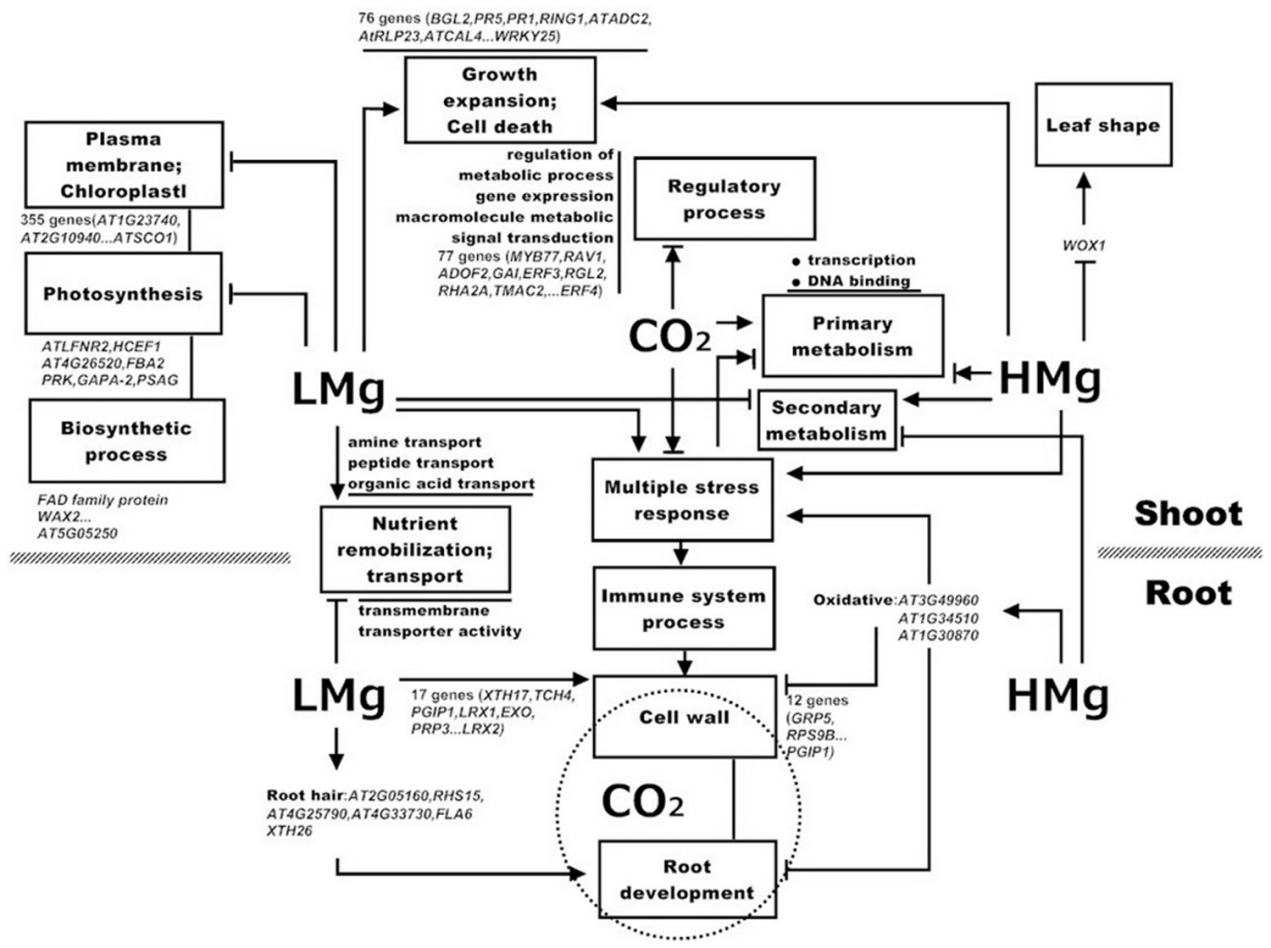
Overview of CO_2_-Mg regulated transcriptome responses in shoots and roots. Putative regulated processes are enclosed in boxes. For illustration, some representative genes are given in italics. Solid arrows indicate links established in the enhanced regulation and dashed circle denote moderation of elevated CO_2_ on cell wall in the root. T bars, Inhibition. Different colors indicate different regulatory signals. Abbreviations: LMg, low Mg, HMg, High Mg.

We proposed a model to show how the combined effect of elevated CO_2_ and Mg levels regulates the transcriptome profile in *Arabidopsis* (Fig 9). This model is mainly based on our analysis of gene functions and pathways. In shoots, both low Mg and high Mg induced but elevated CO_2_ restrained multiple-stress response and cell death, and subsequently immune system process. In addition, CO_2_ and Mg stresses commonly influenced the primary and secondary metabolism, displaying an inhibitory effect of low Mg but a promoting effect of both high Mg and elevated CO_2_. Moreover, low Mg specially inhibited photosynthesis including plasma-membrane and chloroplast as well as some related biosynthetic process, whereas high Mg altered leaf shape. On the other hand, low Mg enhanced but high Mg decreased cell wall synthesis and root-hair cell differentiation (e.g. *ATXTH17*, *ATXTH 26*, *LEUCINE-RICH REPEAT/EXTENSIN 1*, *EXORDIUM*, *PRP3*, *POLYGALACTURONASE INHIBITING PROTEIN 1* and *GLYCINE-RICH PROTEIN 5*) (Fig 9). Under both low Mg and high Mg conditions, some DEGs were elevated in those involved in immune system functions, in particular, functions related to cell death, and toxin catabolic and metabolic process and defense response. Importantly, elevated CO_2_ mitigates the impact of low or high Mg on cell wall and root development, which is exactly the interaction nodes of combined treatment of CO_2_ and Mg. However, the current model still remains incomplete because the integrated output of hormonal cross-talks in response to the combined treatment of CO_2_ and Mg is complex due to the synergistic, additive or antagonistic effects on signaling pathways. These findings provide new insights in the interaction between CO_2_ and Mg nutrition on molecular physiology of the plants, which will help to design the novel metabolic engineering strategies to crops (Brassica as relatives to the model species) with Mg deficiency/excess in crop plants under elevated CO_2_.

## Supporting Information

S1 Table List of transcriptomic data under individual vs. combinatorial treatments of elevated CO_2_ and Mg levels. (XLS)

S2 Table The Pearson correlation coefficients of sample pairs. (XLSX)

S3 Table List of differentially regulated genes expression in venn diagram. (XLS)

S4 Table List of primers used for the reverse transcription quantitative polymerase chain reaction (qPCR) assay and transcriptomic data of reconfirmed genes. (XLS)

S5 Table Gene ontology analysis of differentially expressed genes (DEGs). (XLS)

S6 Table Gene ontology analysis of hierarchical clusters as shown in Figs 5 and 6. (XLS)
